# Validation of a Liquid Chromatography-Electrospray Ionization-Tandem Mass Spectrometry Method for Determination of All-*Trans* Retinoic Acid in Human Plasma and Its Application to a Bioequivalence Study

**DOI:** 10.3390/molecules19011189

**Published:** 2014-01-17

**Authors:** Jing-Bo Peng, Chen-Hui Luo, Yi-Cheng Wang, Wei-Hua Huang, Yao Chen, Hong-Hao Zhou, Zhi-Rong Tan

**Affiliations:** 1 Institute of Clinical Pharmacology, Central South University, Changsha, Hunan 410078, China; E-Mails: jingbopeng@csu.edu.cn (J.-B.P.); leewanaimuting@sohu.com (Y.-C.W.); endeavor34852@aliyun.com (W.-H.H.); cbohua@163.com (Y.C.); hhzhou2003@163.com (H.-H.Z.); 2 Hunan Provincial Tumor Hospital, The Affiliated Tumor Hospital of Xiangya Medical School of Central South University, Changsha, Hunan 410013, China; E-Mail: lchhr0107@163.com

**Keywords:** all-*trans* retinoic acid (ATRA), LC-MS-MS, liquid-liquid extraction, bioequivalence

## Abstract

A sensitive, reliable and specific LC-MS-MS method was developed and validated for the identification and quantitation of all-*trans* retinoic acid (ATRA) in human plasma. Acitretin was used as the internal standard (IS). After liquid-liquid extraction of 500 μL plasma with methyl *tert*-butyl ether (MTBE), ATRA and the IS were chromatographed on a HyPURITY C18 column (150 mm × 2.1 mm, 5 μm) with the column temperature set at 40 °C. The mobile phase was consisted of 40% phase A (MTBE–methanol–acetic acid, 50:50:0.5, v/v) and 60% phase B (water–methanol–acetic acid, 50:50:0.5, v/v) with a flow rate of 0.3 mL/min. The API 4000 triple quadrupole mass spectrometer was operated in multiple reaction monitoring (MRM) mode via the positive electrospray ionization interface using the transition *m/z* 301.4 → 123.1 for ATRA and *m/z* 326.9 → 177.1 for IS, respectively. The calibration curve was linear over the range of 0.45–217.00 ng/mL (r ≥ 0.999) with a lower limit of quantitation (LLOQ) of 0.45 ng/mL. The intra- and inter-day precisions values were below 8% relative standard deviation and the accuracy was from 98.98% to 106.19% in terms of relative error. The validated method was successfully applied in a bioequivalence study of ATRA in Chinese healthy volunteers.

## 1. Introduction

Acute promyelocytic leukemia (APL), classified as acute myeloid leukemia (AML) subtype M3 is characterized by a chromosomal translocation involving the retinoic acid receptor alpha (RARα or RARA) gene, which is distinct from other forms of AML in its responsiveness to all-*trans* retinoic acid (ATRA) therapy. ATRA plus chemotherapy has achieved in 5-year disease free survival exceeding 70% for APL care [1]. As an analogue of vitamin A, ATRA is able to induce apoptosis and differentiation of immature promyelocytes and decrease the risk of bleeding in patients with APL [2]. In addition to its pharmacodynamics, the pharmacokinetics of ATRA plays a significant role for its efficacy in the treatment of APL. ATRA was well absorbed after oral administration and the plasma half-life of ATRA was quite short (<1 h), while ATRA was oxidized to 4-oxo-ATRA and isomerized to 13-*cis* retinoic acid. Continued oral doses of ATRA were associated with a significant decrease in both the area under the concentration-time curve and the plasma peak levels (*p* = 0.004 and 0.01, respectively) when tested after 2–6 weeks of treatment [3]. No accumulation was observed after multiple doses and ATRA was not retained in tissues. Despite its general efficacy, 10% to 20% patients with APL still relapse after therapy with ATRA. The differential ATRA sensitivity might be related to the CYP26 gene expression which was known to be involved in retinoic acid catabolism [4,5]. Although the pharmacokinetic parameters of ATRA in health volunteers have been described previously [6,7], no report has been performed for Chinese subjects.

As ATRA is rapidly isomerized and degraded by oxidants and light, this makes the stability of method critical and requires careful chromatography. Considerable attention must be given to the sample collection, preparation and storage conditions. Existing analytical methods for retinoid analysis using various analytical techniques, such as high performance liquid chromatography-ultraviolet detection (HPLC-UV) [8], gas chromatography-mass spectrometry (GC-MS) [9], liquid chromatography-mass spectrometry (LC-MS) [10,11] have been reported. However, HPLC-UV and GC-MS methods suffer from a number of disadvantages, such as poor selectivity and complicated and prolonged sample preparation. Especially, the re-dissolved analyte showed poor stability over sample preparation in the published LC-MS method when processed in our method validation [10,11]. Thus, our objective was to develop a sensitive, reliable and specific HPLC-MS-MS method with simple LLE extraction preparation and a 15 min run time, for the quantification of ATRA in human plasma. The analytical method described in this study was fully validated and successfully used to evaluate the bioequivalence of two pharmaceutical formulations of ATRA capsules in healthy Chinese male volunteers.

## 2. Results and Discussion

### 2.1. Method Development and Optimization

#### 2.1.1. Optimization of Sample Preparation

A sample preparation method should reduce interferences from the biological sample and not affect the sensitivity and robustness of the assay. Liquid-liquid extraction (LLE) and protein precipitation (PPT) were exploited for separating ATRA from plasma samples. Compared with PPT, LLE gave both high extraction efficacy and less interference. MTBE, diethyl ether, *n*-hexane–dichloromethane–isopropanol (10:20:1, v/v/v) were all investigated as extraction solvents, and MTBE was finally employed because of its higher extraction recovery (>75%) to ATRA and IS. Additionally, different reconstitution solutions of the residue were also investigated. Compared to initial mobile phase solvent, reconstituted with mixed solvent (formaldehyde–dimethyl formamide, 50:50, v/v) achieved excellent sample stability.

#### 2.1.2. Optimization of the LC-MS-MS Condition

**Figure 1:**
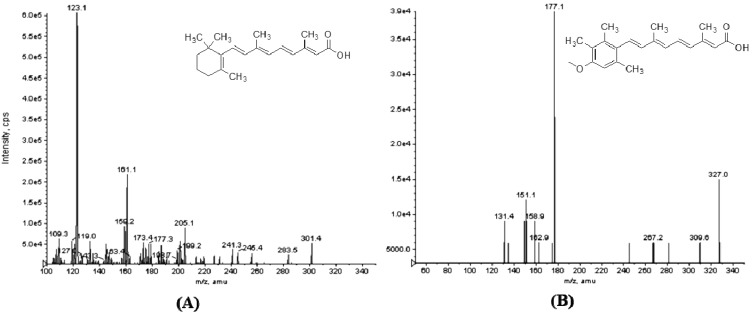
Product ion mass spectra of (A) ATRA (*m/z* 301.4 → 123.1) and (B) acitretin (IS, *m/z* 326.9 → 177.1) in positive ionization mode.

Mass spectrometry operation parameters were carefully optimized for the determination of ATRA and IS. Compared to the atmospheric pressure ionization (APCI) source, ESI was selected because it offered better efficiency of ionization for ATRA. Standard solutions of ATRA and IS were directly infused along with mobile phase and the signal intensity of positive ions were stronger than for negative ions, which indicated that the positive mode was much more sensitive. In the precursor ion full-scan spectra, the most abundant ions were protonated [M+H]^+^ molecules at *m/z* 301.4 and 326.9 for ATRA and the IS, respectively. The product ion spectra of the parent ions showed high abundant daughter fragments at *m/z* 123.3 and 177.1 for ATRA and the IS (Figure 1).

Therefore, these ions were chosen as precursor→product ion transitions for the multiple-reaction-monitoring (MRM) mode. The collision energy in the product MS-MS mode was optimized for maximum response of the fragmentation of analytes, and the optimal values were found to be 23, and 16 eV for ATRA and the IS, respectively.

ATRA was endogenously present in the human body with isomeric metabolites (*i.e.*, *iso*-ATRA and 9-oxoretinoic acid). The development of the chromatographic conditions was focused on chromatographic separation rather than on short retention times. The organic solvent compositions and percentage in the mobile phase were investigated for separating ATRA from isomeric metabolites. Finally, a mobile phase of 40% phase A (MTBE-methanol-acetic acid, 50:50:0.5, v/v) and 60% phase B (water-methanol-acetic acid, 50:50:0.5, v/v) was employed to separate and accurately measure the concentrations of ATRA in plasma with good separation and lower matrix effect. Under the optimal conditions, a flow rate of 0.3 mL/min with the total run time of 15 min for each sample was employed.

### 2.2. Method Validation

#### 2.2.1. Selectivity

**Figure 2:**
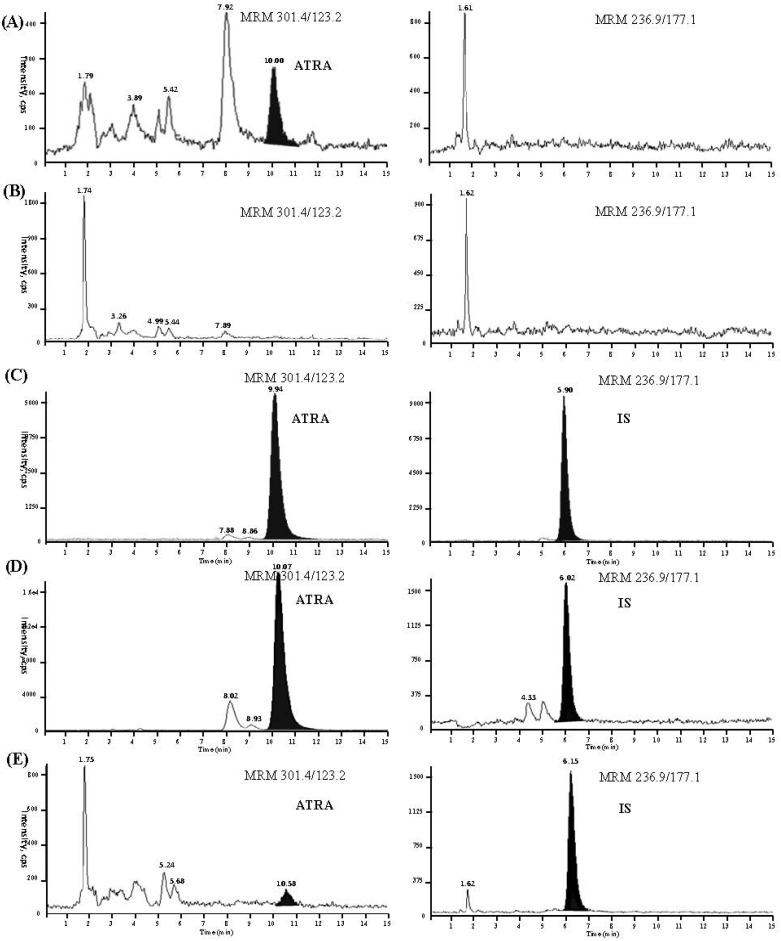
The typical MRM chromatograms of a blank plasma (**A**), a blank plasma with sunshine exposure treatment (**B**), a plasma sample with sunshine exposure treatment spiked with ATRA (108.5 ng/mL) and IS (114.4 ng/mL) (**C**), a plasma sample from a healthy volunteer 2 h after oral administration of 20 mg ATRA (**D**), and ATRA at the LLOQ with IS (**E**).

The LC-MS-MS method has a high selectivity due to good separation of ATRA, and since only ions derived from the analytes of interest were monitored. Figure 2A–D show the typical MRM chromatograms of a blank plasma sample, a blank plasma sample with sunshine exposure treatment, a plasma sample with sunshine exposure treatment spiked with ATRA (108.50 ng/mL) and IS (114.40 ng/mL), and a plasma sample from a healthy volunteer 2 h after oral administration of 20 mg ATRA. As shown, no interference from endogenous substance at the retention times of ATRA and the IS was observed. Retention times were approximately 10.0 min and 5.9 min for ATRA and the IS, respectively.

#### 2.2.2. Linearity and Lower Limit of Quantification

A calibration curve was conducted on each validation day. The peak area ratio of ATRA to IS in human plasma varied linearly over the concentration range of 0.45–217.00 ng/mL (r ≥ 0.999). The mean regression equation from replicate calibration curves from three consecutive days was *y* = 0.0761*x* + 2.651 × 10^−3^ (*r* = 0.999), where *y* is the peak-area ratio of ATRA to IS and *x* is the concentration of ATRA in plasma, and the linear least-squares regression with a weighing index of 1/*x*. The lower limit of quantitation (LLOQ) of this method was 0.45 ng/mL at which the signal-to-noise ratio (S/N) of ATRA was higher than ten (Figure 2E). The precision expressed as RSD was 5.44% (n = 5), and the accuracy expressed as RE was 5.22% (n = 5).

#### 2.2.3. Precision and Accuracy

Data for intra- and inter-day precision and accuracy for the determination of ATRA from QC samples are shown in Table 1. For the devised assay, intra-day RSD was <5.90%, and accuracy ranged from 98.98% to 106.19%. The inter-day RSD was <7.46%, and accuracy from 102.39% to 105.48%. The data of this method was well within the ranges of precision (%) and accuracy (%) specified by the FDA for bio-analytical method validation [12].

**Table 1:** Intra- and inter-day precision and accuracy for LC-MS/MS assay of ATRA in human plasma.

Theoretical conc. (ng/mL)	Intra-batch (n = 5)			Inter-batch (n = 15)		
	Detected conc. (mean ± SD) (ng/mL)	Accuracy (%)	Precision (%)	Detected conc. (mean ± SD) (ng/mL)	Accuracy (%)	Precision (%)
0.90	0.96 ± 0.06	106.19	5.90	0.95 ± 0.07	105.48	7.46
12.06	11.94 ± 0.35	98.98	2.95	12.35 ± 0.65	102.39	5.30
173.60	176.50 ± 5.90	101.66	3.34	178.99 ± 8.07	103.10	4.51

#### 2.2.4. Extraction Recovery and Matrix Effect

Data for extraction recovery and matrix effect for the determination of ATRA and the IS from QC samples are shown in Table 2. The extraction recoveries of ATRA from QC samples ranged from 75.63% to 81.61%. The extraction recovery of the IS was 76.27% ± 11.41%. Thus, the consistency in recoveries of ATRA and the IS supported that the extraction procedure was available for a large numbers of samples analysis. The matrix effects for ATRA from QC samples ranged from 74.33% to 86.38%, indicating that no endogenous co-elution influenced the ionization of ATRA.

**Table 2:** Recovery and matrix effect of ATRA and the IS using liquid-liquid extraction.

Analyte	Concentration (ng/mL)	Recovery (C/B, %)	Matrix effect (B/A, %)
ATRA	0.90	81.61 ± 8.66	74.33 ± 4.88
	12.06	81.40 ± 7.77	77.64 ± 3.39
	173.60	75.63 ± 1.63	86.38 ± 6.65
IS	114.40	76.27 ± 11.41	92.24 ± 2.42

A, mean area response of three replicate samples for ATRA/IS prepared in mobile phase; B, mean area response of three replicate samples for ATRA/IS prepared by spiking in extracted blank plasma; C, mean area response of three replicate samples for ATRA/IS prepared by spiking before extraction.

#### 2.2.5. Stability

The stability results under certain storage conditions are summarized in Table 3. No chemical or biological degradation of ATRA were observed during sample storage (in plasma at room temperature for 4 h, in plasma at −40 °C for 65 days), three freeze-thaw cycles and post-treatment (in the reconstituted extraction at room temperature for 48 h). All the samples displayed 85%–115% recoveries after various stability tests, which indicated that the method was applicable for routine analysis.

### 2.3. Application to Biological Samples

**Figure 3:**
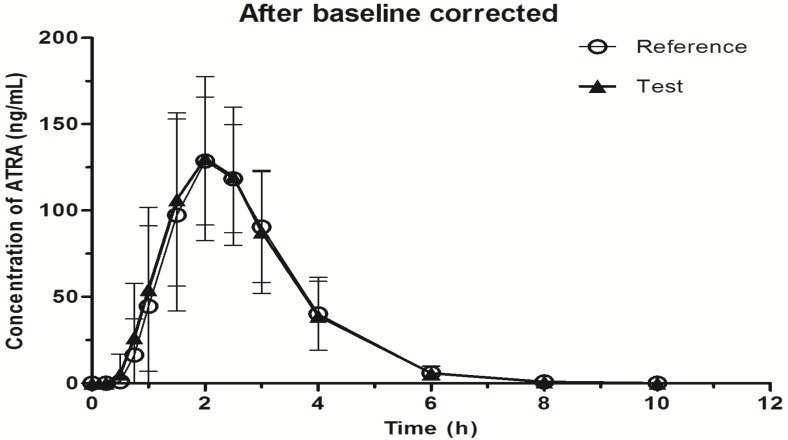
Comparison of the mean plasma concentration time curves of ATRA (test *vs*. reference) from 29 volunteers after baseline corrected.

The validated LC-MS-MS method was applied to a bioequivalence study of two formulations of ATRA produced by Chongqing HuaPont Pharmaceutical Co., Ltd. (Chongqing, China). Pharmacokinetic and statistical analyses were conducted on both baseline corrected and un-corrected data with mean concentration of pre-dose time points collected at −24, −20, −12 and 0 h. The calculated pharmacokinetic parameters were shown in Table 4, and the mean concentration time curve of ATRA was shown in Figure 3.

**Table 3:** Stability results for ATRA under different conditions (n = 5).

Storage conditions	Theoretical conc. (ng/mL)	Detected conc. (mean ± SD) (ng/mL)	RSD (%)	RE (%)
Freeze-thaw (three cycles, −40 °C)	0.90	0.87 ± 0.07	8.57	−3.56
	12.06	12.14 ± 0.33	2.73	0.70
	173.60	183.60 ± 13.65	7.44	5.78
Auto sampler (48 h, 25 °C)	0.90	0.99 ± 0.03	3.23	9.17
	12.06	12.99 ± 0.55	4.25	7.71
	173.60	180.10 ± 15.78	8.76	3.76
Short-term (4 h, 25 °C)	0.90	1.00 ± 0.03	2.97	10.72
	12.06	11.88 ± 0.17	1.42	−1.53
	173.60	167.10 ± 10.55	6.31	−3.74
Long-term (65 days, −40 °C)	0.90	0.99 ± 0.04	4.56	9.21
	12.06	11.28 ± 0.39	3.43	−6.50
	173.60	171.20 ± 11.83	6.91	−1.36

**Table 4:** The main pharmacokinetic parameters of ATRA after single oral dose of 20 mg to healthy volunteers (before and after baseline corrected).

Parameter	Before baseline corrected		After baseline corrected	
	Reference drug	Test drug	Reference drug	Test drug
T_1/2_ (h)	1.62 ± 3.68	1.32 ± 2.24	0.79 ± 0.47	0.71 ± 0.12
T_max_ (h)	2.10 ± 0.40	2.10 ± 0.30	2.05 ± 0.36	2.07 ± 0.29
C_max_ (ng/mL)	141.75 ±31.64	142.52 ± 34.09	140.79 ± 31.64	141.59 ± 34.09
AUC_0→t_ (ng∙h/mL)	343.95 ± 100.7	352.67 ± 121.78	334.11 ± 100.32	343.19 ± 121.56
AUC_0→∞_ (ng∙h/mL)	345.67 ± 98.96	353.91 ± 120.71	335.09 ± 100.26	344.11 ± 121.58

Bioequivalence of two formulations of ATRA was analyzed by means of variance (ANOVA) using log-transformed data for crossover design and calculating 90% confidence intervals (CI) of the ratio of test/reference. The formulations were considered bioequivalent because the difference between two compared pharmacokinetic parameters was found statistically insignificant (*p* > 0.05). Furthermore, the 90% CI for the ratios of test and reference formulation derived from the analysis of log-transformed pharmacokinetic parameters (baseline uncorrected and corrected), AUC_0→t_, AUC_0→∞_ and C_max_ were 94.2%~106.1% and 93.9%~106.3%, 94.2%~106.0% and 93.9%~106.3%, 95.8%~104.4% and 95.8%~104.4%, respectively. The test ATRA soft capsule was considered to be bioequivalent to the reference ATRA capsule.

## 3. Experimental

### 3.1. Chemicals and Reagents

Reference standards of ATRA (purity: 98.6%) and acitretin (purity: 100.0%) were kindly provided by Chongqing HuaPont Pharmaceutical Co., Ltd. (Chongqing, China). ATRA test soft capsules and ATRA reference capsules were supplied and identified by Chongqing HuaPont Pharmaceutical Co., Ltd. Methanol, acetic acid and methyl *tert*-butyl ether (MTBE) of LC grade were obtained from CNW (CNW Technologies GmbH, Düsseldorf, Germany), other reagents were of analytical grade, and all water used was Milli-Q grade.

### 3.2. Liquid Chromatographic Conditions

The chromatographic separation was performed on a Waters AcQuitry series Ultra Performance (Milford, MA, USA) system. A HyPURITY C18 (150 mm × 2.1 mm, 5 µm) column from Thermo (Waltham Mass, MD, USA) was used for separation maintained at 40 °C. The mobile phase was consisted of 40% phase A (methyl *tert*-butyl ether–methanol–acetic acid, 50:50:0.5, v/v) and 60% phase B (water–methanol–acetic acid, 50:50:0.5, v/v) with a flow rate of 0.3 mL/min. Prior to analysis, the column was equilibrated with the mobile phase at a flow rate of 0.3 mL/min for about 15 min. The auto-sampler was maintained at 10 °C and 10 µL of sample solution was injected with partial loop mode. The total running time was 15 min for each injection.

### 3.3. Mass Spectrometry Conditions

An API4000 triple quadrupole mass spectrometer from ABI (Thornhill, ON, Canada) equipped with an electrospray ionization (ESI) interface was operated for analytical determination. The ESI source was set in positive ionization mode. The quantitation was conducted using multiple reaction monitoring (MRM) of the transitions of *m/z* 301.4 → 123.1 for ATRA and *m/z* 326.9 → 177.1 for IS, with a dwell time of 250 ms. The optimal MS parameters were as follows: the ion spray voltage was 5.5 kV; ion source temperature of GS2 was set at 500 °C. Nebulizing gas of nitrogen (GS1), turbo spray gas (GS2), and curtain gas (CUR) were 30, 30 and 6.0 Psi, respectively. Unit resolution was set for both Q1 and Q3 mass detection, and the collision energy was set at 23 and 16 eV for ATRA and IS, respectively. Data acquisition and processing were accomplished using Analyte version 1.4.2 software (Applied Biosysems/MDS-Sciex, Concord, ON, Canada).

### 3.4. Preparation of Standard and Quality Control Samples

Stock standard solutions of ATRA and IS were both prepared in methanol at the concentration of 1.085 mg/mL and 1.144 mg/mL, respectively. The stock solution of ATRA was then serially diluted with methanol to provide working standard solutions of desired concentrations. An IS working solution of 114.40 ng/mL was obtained by diluting the stock solution of IS with methonal–water (50:50, v/v). All the solutions were stored at 4 °C until required. It’s worth mentioning the experimental process was performed in the dark with red light, and ATRA was kept in amber containers for the experiment.

As ATRA was very sensitive to light, blank plasma with sunshine exposure treatment for 6 h was used for the preparation of calibration standard samples and quality control (QC) samples to reduce the interference of endogenous ATRA. 50 μL of the appropriate working solutions were put into glass tubes, after evaporated to dryness under nitrogen, 500 μL of blank plasma was spiked to give concentrations of 0.45, 0.90, 1.81, 5.42, 12.06, 36.17, 108.50, and 217.00 ng/mL. QC samples in the plasma were prepared in a similar way at low, medium and high concentrations of 0.90, 12.06, 173.60 ng/mL.

### 3.5. Plasma Sample Preparation

In the analyses, plasma ATRA calibrators, QCs and human samples were prepared using the following protocol. A 500 μL aliquot of sample plasma in a disposable Eppendorf tube, 100 μL of IS working solution (114.40 ng/mL) was added. The mixture was vortexed for 30 s and extracted with 2 mL MTBE by vortex mixing for 1 min and centrifugation at 4,000 rpm for 10 min. The supernatant was separated and evaporated to dryness at 37 °C under a gentle stream of nitrogen. The residue was then reconstituted in 200 μL of formaldehyde-dimethyl formamide (50:50, v/v), followed by vortexing and centrifugation at 13,000 rpm for 10 min before analysis. An aliquot of 10 µL was injected into the LC-MS-MS system.

### 3.6. Method Validation

The method was validated according to the Food and Drug Administration (FDA) guidelines [12] on selectivity, linearity, sensitivity, precision, accuracy, extraction recovery, matrix effect, and stability. The validation runs were conducted on three consecutive days. The peak area ratios of ATRA to the IS of QC samples were interpolated from the calibration curve on the same day to give the concentrations of ATRA. The results from QC samples for three runs were used to evaluate the precision, accuracy and stability of the method developed. 

#### 3.6.1. Selectivity

The selectivity was evaluated by comparing the chromatograms of six different blank plasma with sunshine exposure treatment from six subjects to those of corresponding plasma spiked with ATRA and IS, and those of plasma samples from volunteers after oral administration of ATRA soft capsules. 

#### 3.6.2. Linearity and Lower Limit of Quantification

To evaluate the linearity, calibration standards of ATRA at eight concentrations over the range of 0.45–217.00 ng/mL were extracted and assayed in three separate runs. The calibration curves were constructed by plotting the peak-area ratio of ATRA to IS versus the concentrations (*x*) of ATRA, using weighted (1/*x*) least squares linear regression. The lower limit of quantification (LLOQ) for ATRA was defined as the lowest concentration of the calibration curve and could be quantitatively determined with an acceptable precision (relative standard deviation, RSD) and accuracy (relative error, RE). It was determined by using five replicates, and the precision (below 20%) and accuracy (within 80%–120%) were defined.

#### 3.6.3. Precision and Accuracy

The intra-day and inter-day precision and accuracy of the method were evaluated by analyzing five replicate of QC samples at three concentrations on the same day or on the three consecutive days. The concentration of each sample was calculated using standard curve prepared and analyzed on the same day. The precision was determined as the RSD and the accuracy as the RE.

#### 3.6.4. Extraction Recovery and Matrix Effect

The extraction recovery was assessed by comparison of the peak areas of the spiked ATRA/IS extracted from plasma samples with those from post-extracted blank plasma spiked at corresponding concentrations. This procedure was repeated (n = 6) at three QC concentration levels of 0.90, 12.06, and 173.60 ng/mL. The matrix effect of method was measured by comparing the peak areas of analytes spiked post-extraction with that of pure reference standard solutions at three concentration levels dried directly.

#### 3.6.5. Stability

The stability of ATRA in human plasma was evaluated by analyzing three replicates of plasma samples at concentrations of 0.90, 12.06, and 173.60 ng/mL during the sample storage and processing procedures. The short-term stability was evaluated after the exposure of the spiked samples at room temperature for 4 h, and the ready-to-inject samples (after extraction, in the mobile phase) at room temperature for 48 h. The long-term stability was estimated by processing the standard spiked plasma samples after the storage at −40 °C for 65 days. The freeze-thaw stability was conducted by allowing QC samples to undergo three complete freeze/thaw cycles (−40 to 25 °C) before extraction. The concentrations of QC samples were calculated by using calibration curves of freshly prepared standards. The stability of stock solutions of ATRA (108.50 ng/mL) and the IS (114.40 ng/mL) in methanol at 4 °C for 65 days, and at room temperature for 6 h were also assessed. The mean peak areas of three replicates of ATRA and the IS samples were compared with those from freshly prepared solutions at the same concentration.

### 3.7. Bioequivalence Study

The validated LC-MS-MS method was applied to a bioequivalence study of two formulations of ATRA in which 29 healthy male volunteers were enrolled. The pharmacokinetic study was approved by the medical Ethics Committee of Hunan Xiangya Hospital (Hunan, China). Informed consent was obtained from all volunteers after explaining the aims and risks of the study according to the principles of the Declaration of Helsinki. All volunteers were alcohol, tobacco, and drug free and had a body mass index (BMI) between 19 and 24. Twenty-nine volunteers were randomized into two groups, and oral administrated a single dose of 20 mg ATRA soft capsule or ATRA capsule with 200 mL of water after a 12-h overnight fast, respectively. An indwelling cannula was placed in one arm for blood sampling. Blood samples were collected prior to drug administration (−24, −20, −12, 0 h), and at 0.25, 0.5, 0.75, 1, 1.5, 2.5, 3, 4, 6, 8, and 10 h after drug administration. The blood samples were rapidly centrifuged at 3,000 rpm for 10 min to separate the plasma. It’s worth to mention that the blood sampling and plasma preparation procedure were conducted in dark with red light. The collected plasma samples were stored at −40 °C until analysis. 

The pharmacokinetic parameters were analyzed by non-compartmental assessment of data using Drug and Statistic (DAS) software (version 3.2.2, Clinical Drug Evaluation Center, Wannan medical College, Anhui, China). Bioequivalence of two formulations was estimated by means of analysis of variance (ANOVA). The maximum plasma concentration (*C*_max_) and their time of occurrence (*T*_max_) were noted directly from the measured data. The elimination rate constant (*k*_e_) was calculated by log-linear regression of concentrations observed during the terminal phase of elimination, and the elimination half-life (*t*_1/2_) was then calculated as 0.693/*k*_e_. The area under the plasma concentration-time curve (AUC_0–t_) to the last measurable plasma concentration (*C*_t_) was calculated with the linear trapezoidal rule. The area under the plasma concentration-time curve to time infinity (AUC_0–∞_) was calculated as AUC_0–t_ + *C*_t_/*k*_e_.

## 4. Conclusions

A reliable, selective and specific liquid chromatography tandem mass spectrometric method for determination of ATRA in human plasma has been developed and validated. Samples were isolated by LLE, separated on a C18 column and quantified by LC-MS-MS. The assay exhibited adequate sensitivity, acceptable precision and excellent stability for the quantification of ATRA in human plasma resulting from the pharmacokinetic, bioavailability or bioequivalence studies of ATRA. In addition, when compared with previous studies, the developed method exhibited excellent stability and selectivity for analyzing large samples in pharmacokinetic studies of ATRA. The method has been successfully applied to a bioequivalence study of ATRA in healthy Chinese male volunteers. 
